# The Effect of Y, La, Lu, and K on the Segregation and Cohesion of Tungsten Grain Boundaries

**DOI:** 10.3390/ma19214547

**Published:** 2026-10-28

**Authors:** Mingyu Wu, Chengzhen Miao, Nanxi Zhao, Kaige Shen, Yujuan Zhang, Yan Zhao, Yanfei Qi, Changchun Ge

**Affiliations:** 1 Institute of Nuclear Materials, School of Materials Science and Engineering, University of Science and Technology Beijing (USTB), Beijing 100083, China; 2 College of Science, North China University of Science and Technology (NCST), Tangshan 063210, China; 3 College of Metallurgy and Energy, North China University of Science and Technology (NCST), Tangshan 063210, China

**Keywords:** tungsten grain boundary, strengthening grain boundary, rare earth, potassium, first-principles calculation

## Abstract

We have systematically analyzed the solution and aggregation characteristics of yttrium (Y), lanthanum (La), lutetium (Lu) and potassium (K) on symmetrically inclined tungsten (W) grain boundary (GB) Ʃ5(310)/[001] by first principles calculation. Furthermore, the influence mechanism of Y, La Lu and K on the strength of W GB was analyzed. K/La and Y/Lu exhibit two different evolution characteristics in the W GB region. The calculation results show that Y/Lu preferentially occupies the substitution site (V_0_) on the W GB plane, and the solution energy of Y/Lu at the substitution site increases with distance from the W GB plane. However, the most stable occupation sites of La/K in the W GB region are the sites (V_1_) nearest to the W GB plane. The electronic structure analysis showed that the segregation energies of Y, La Lu and K on the W GB were positively correlated with the number of electron transfer. The existence of K/La in the W GB region promotes the dissolution of subsequent atoms, while the existence of Y/Lu atoms has little effect on the dissolution of subsequent atoms. In terms of mechanical properties, the addition of alloying elements significantly improves the fracture energy of W GB, and the Y and Lu effectively increase the fracture energy to 5.602 J/m^2^ and 5.687 J/m^2^, which is higher than the 5.185 J/m^2^ of clean W GB. This strengthening mechanism is mainly attributed to the strong metal bond between W-Y and W-Lu. Although the fracture energy of La and K is also higher than that of clean W GB—5.465 J/m^2^ and 5.335 J/m^2^, respectively—the strength improvement mainly depends on the action of La-La and K-K metal bonds.

## 1. Introduction

Energy is the cornerstone of modern social operation and development, and its significance to human survival and progress is undeniable. As the global population expands and economies advance, the demand for energy continues to rise. Controlled thermonuclear fusion is regarded as the most promising clean energy source, which barely causes environmental issues such as radioactive pollution [1]. Its fuel, deuterium and tritium, exists in seawater in abundant quantities. The deuterium–tritium fusion reaction releases enormous amounts of energy, and the available fuel reserves on Earth are expected to last for over 30 million years. Therefore, controlled thermonuclear fusion is recognized as a primary approach to effectively addressing the energy demands of humanity in the future [2,3]. Ensuring the reliability and stability of energy supplies is crucial, which is why humanity is beginning to utilize all forms of energy, including highly efficient nuclear energy.

Tungsten (W) has a high melting point, low sputtering and extremely low hydrogen retention, making it an ideal choice as a plasma wall material for fusion reactors [4,5,6]. However, pure tungsten has significant room temperature brittleness and irradiation-induced brittleness, which limits its application [7,8,9]. Therefore, enhancing the toughness and radiation resistance of W-based alloys has emerged as a critical scientific challenge in the research of fusion materials [10]. In order to better use W materials in nuclear fusion reactor materials, many scientists have carried out continuous exploration and research. At present, the two main ways to improve the toughness of W-based alloys are microalloying and W GB strengthening. Microalloying refers to the addition of beneficial alloying elements to effectively improve the strength and toughness of the material [11,12,13]. W GB strengthening refers to improving the brittleness resistance and radiation damage resistance of materials by designing a W GB structure, which is another important improvement direction [14,15,16,17]. Microalloying is an effective way to improve the service properties of fusion W-based alloys [18,19,20]. At the same time, in various alloying strategies, rare earth and K elements are one of the effective ways to improve the toughness of W alloys for nuclear fusion. Adding a certain number of alloying elements to W-based materials can improve their density, toughness, thermal stability and irradiation performance, etc. [21]. Common rare earth elements include Y, La and Lu, which are close to oxygen and tend to aggregate in the W GB region to form oxide particles [11,22]. Therefore, adding rare earth elements to W can increase the toughness of W materials and reduce the cracking of W alloys during nuclear fusion service.

Adding rare earth elements and K elements into W-based materials can improve the service performance of W materials in a fusion environment to a certain extent, but there are still some problems and shortcomings. The addition of Y can refine the grain of W and improve its mechanical properties. The Y oxide is evenly distributed in the W GB region, which makes the grain size of W-Y alloy smaller [23,24]. This is helpful to improve the fracture toughness and service performance of W materials [22]. The addition of La can refine the grain of W and improve its mechanical properties. The addition of K in W enhances the high temperature plasticity. [25,26]. On one hand, the K-bubble in the grain as the dislocation emission source produces a large number of movable dislocations, which improves the plasticity of the material. At the same time, Nano-K foam as the second phase has a dispersion strengthening effect. The K-bubbles can inhibit the formation and accumulation of W interstitial atoms and vacancies during irradiation, thus inhibiting the formation and development of dislocation-type defects [27,28,29]. Wang et al. show that at high temperatures, K vapor in W is forced into unclosed pores, allowing metallurgical pores with higher K content to exist until the end of deformation processing. Through plastic processing, elongated ellipsoidal K-tubes and fine K-bubble columns are formed. K-bubbles have a certain pinning and annihilation effect on W GB, and this effect is more significant at high temperatures [30]. At high temperatures, the K element forms string-like K-bubbles in W, which can pin GBs and improve the service performance of W-based first wall materials in nuclear fusion engineering. The study on the micro-evolution mechanism of rare earth elements and K elements in the W GB region is of great significance for strengthening the mechanical properties of W GB by alloying elements.

In general, the addition of rare earth elements and K has different effects on the mechanical properties of W materials. The specific selection of rare earth elements needs to be evaluated and selected according to the specific application requirements and performance requirements. In order to better understand the influence of rare earth and K on the properties of W materials and determine which element is better from a comprehensive point of view, the solution and segregation characteristics of Y, La, Lu and K in the W GB region were studied by first-principles calculation. The aggregation characteristics of alloying elements in the W GB region are calculated, and the influence of alloying elements on the mechanical properties of W GB is analyzed, which provides a theoretical reference for the development of advanced W-based materials.

## 2. Computational Methods

**Figure 1:**
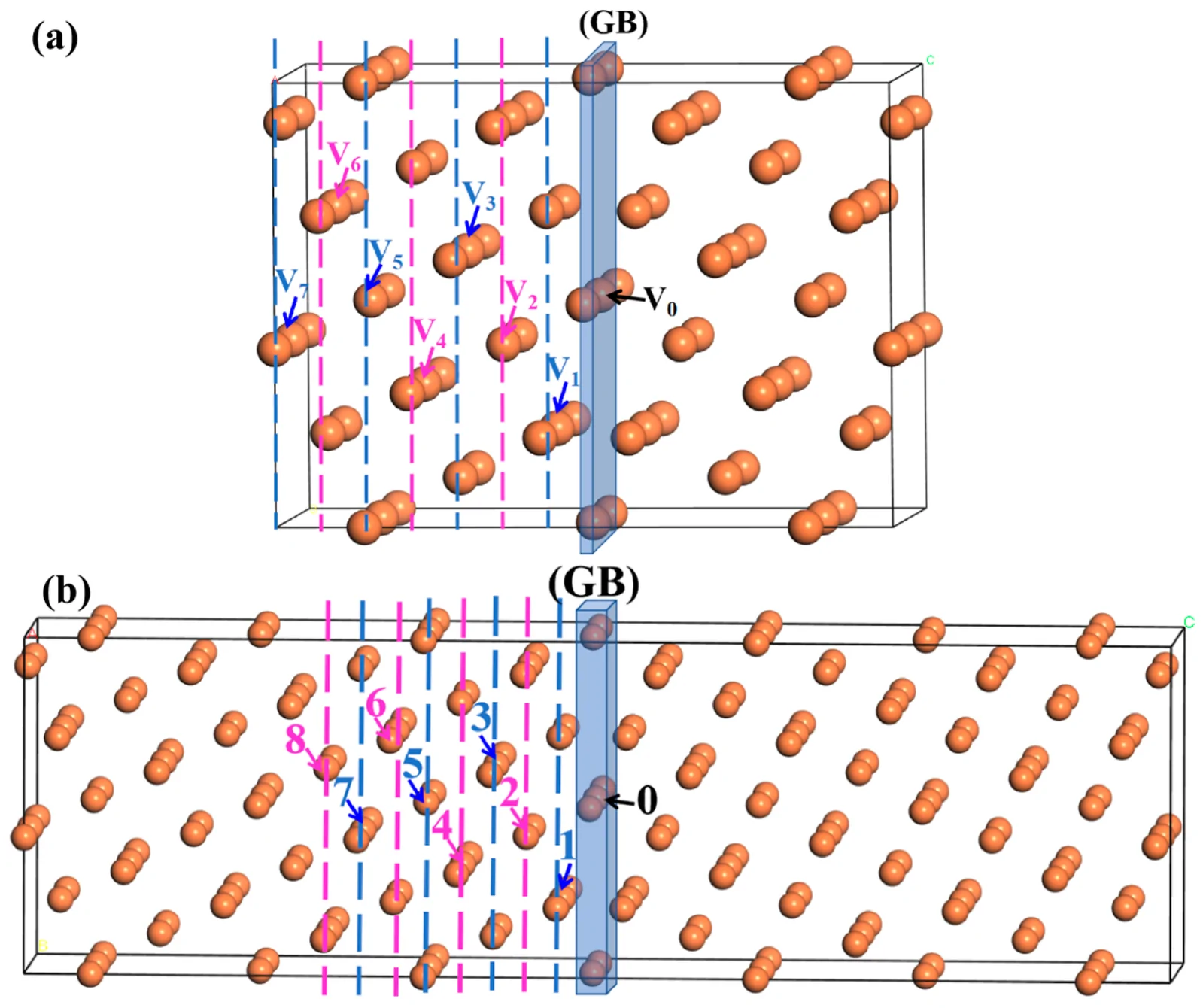
(**a**,**b**) represent Σ5 (310)/[001] W GB containing 56 atoms and 136 atoms respectively. In V_0_–V_7_, 0–8 represent different displacement sites at the dotted line or W GB.

All calculations in this paper were performed using the Vienna Ab initio Simulation Package (VASP.5.4.4), a plane-wave-based density functional theory (DFT) code [31,32]. The generalized gradient approximation (GGA-PBE) was adopted to describe electron–electron interactions, while the projector-augmented wave (PAW) method was used to treat plane-wave functions. The electron arrangement of Y, La and Lu elements is [Kr]4d^1^5s^2^, [Xe]5d^1^6s^2^ and [Xe]4f^14^5d^1^6s^2^, respectively. It should be noted that we use the ordinary PBE pseudopotential. The 4d orbital electrons of La atoms and the 4f orbital electrons of Lu atoms are treated as core electrons. The electron–electron exchange-correlation part is described using the generalized gradient approximation (GGA), with the plane-wave cutoff energy set to 350 eV [33]. The W GB structure was described by the coincident site lattice (CSL) model [34,35]. Specifically, two identical body-centered cubic structures rotate 36.9° around the [001] axis to obtain a symmetrically tilted Σ5(310)/[001] W GB [36,37,38,39]. The Σ5(310)/[001] W GB has clearly obtained the arrangement of atoms through experiments, which is often used to study the segregation of solute atoms in metal materials, and the relationship between the influence of solute atoms on its toughness and brittleness and the evolution of W GB [40]. The occupation of the electronic states is expanded using the Methfessel–Paxton method with a broadening of 0.02 eV. Atomic positions and lattice constants were optimized concurrently until the residual force on each atom was less than 10^−3^ eV/A [41]. The total energy of each supercell is relaxed until the difference is less than 10^−5^ eV. To verify the influence of supercell size on the W GB energy, supercells with 56 and 136 atoms were selected respectively. When using a 56-atom supercell, the K-point grid was selected as 9 × 3 × 1, and when using a 136-atom supercell, the K-point grid was selected as 3 × 3 × 1, as shown in Figure 1. Unless otherwise stated, all the calculated data presented in this chapter were obtained using a 56-atom supercell with the W GB system, where the atomic positions and supercell dimensions were fully relaxed [42]. Due to the existence of periodic boundary conditions, the W supercell contains two W GBs. As shown in Equation (1), the W GB energy *γ* can be obtained from the total energy *E*_g_ of supercells containing W GB, the total energy *E*_s_ of bulk supercells with the same number of atoms, and the cross-sectional area of W GB *S* [43,44]. As shown in Figure 1, the area of the cross-section of the shaded part is S, which includes both the intermediate and boundary boundaries.

(1) $$\gamma =\frac{E_{g}-E_{s}}{S},$$

We calculated the Ʃ5(310)/[001] W GB energies of 2.252 J/m^2^ and 2.282 J/m^2^ using a 56-atom supercell and 136-atom supercell [15], respectively. The W GB energy we calculated is in good agreement with the W GB energy (2.23 J/m^2^) [43,44] obtained from previous experimental studies, indicating that the model and calculation parameter settings we constructed are reasonable.

The solubility energy of a single atom X(Y, La, Lu or K) at the substitution site can be obtained through Formula (2) [4].

(2) $$E_{S}^{sol}=E_{(N-1)W+X}^{T}-E_{NW}^{T}+E_{W}-E_{X},$$

where X represents a Y, La, Lu or K atom; the total energy of the W GB system containing N-1 W atoms and one X atom is represented by $E_{(N-1)W+X}^{T}$; the total energy of the W-GB system containing N W atoms is represented by $E_{NW}^{T}$; and the energies of a single W atom and X atom are represented by $E_{W}$ and $E_{X}$, respectively. The energy of a single atom is calculated by placing the atom in a sufficiently large vacuum box. The solubility energy of a single atom X at the interstitial site can be obtained through Formula (3).

(3) $$E_{I}^{sol}=E_{NW+X}^{T}-E_{NW}^{T}-E_{X},$$

The total energy of N W atoms and one interstitial atom X in the W GB system is represented by $E_{NW+X}^{T}$, and the energy of N W atoms and a single X atom is represented by $E_{NW}^{T}$ and $E_{X}$, respectively.

The segregation energy is a physical quantity that quantitatively describes the driving force of solute atoms migrating from the matrix to W GB. When calculating the segregation energy, the same supercell, lattice vector, and relaxation process are used for both W GB and bulk configurations. The segregation energy can be derived by [43]

(4) $$E^{seg}=E_{GB}^{X}-E_{Bulk},$$

The total energy of solute atoms in the W GB region system is represented by $E_{\mathrm{GB}}^{X}$, and the total energy of solute atoms in the bulk environment system of W GB system is represented by $E_{\mathrm{Bulk}}$.

**Figure 2:**
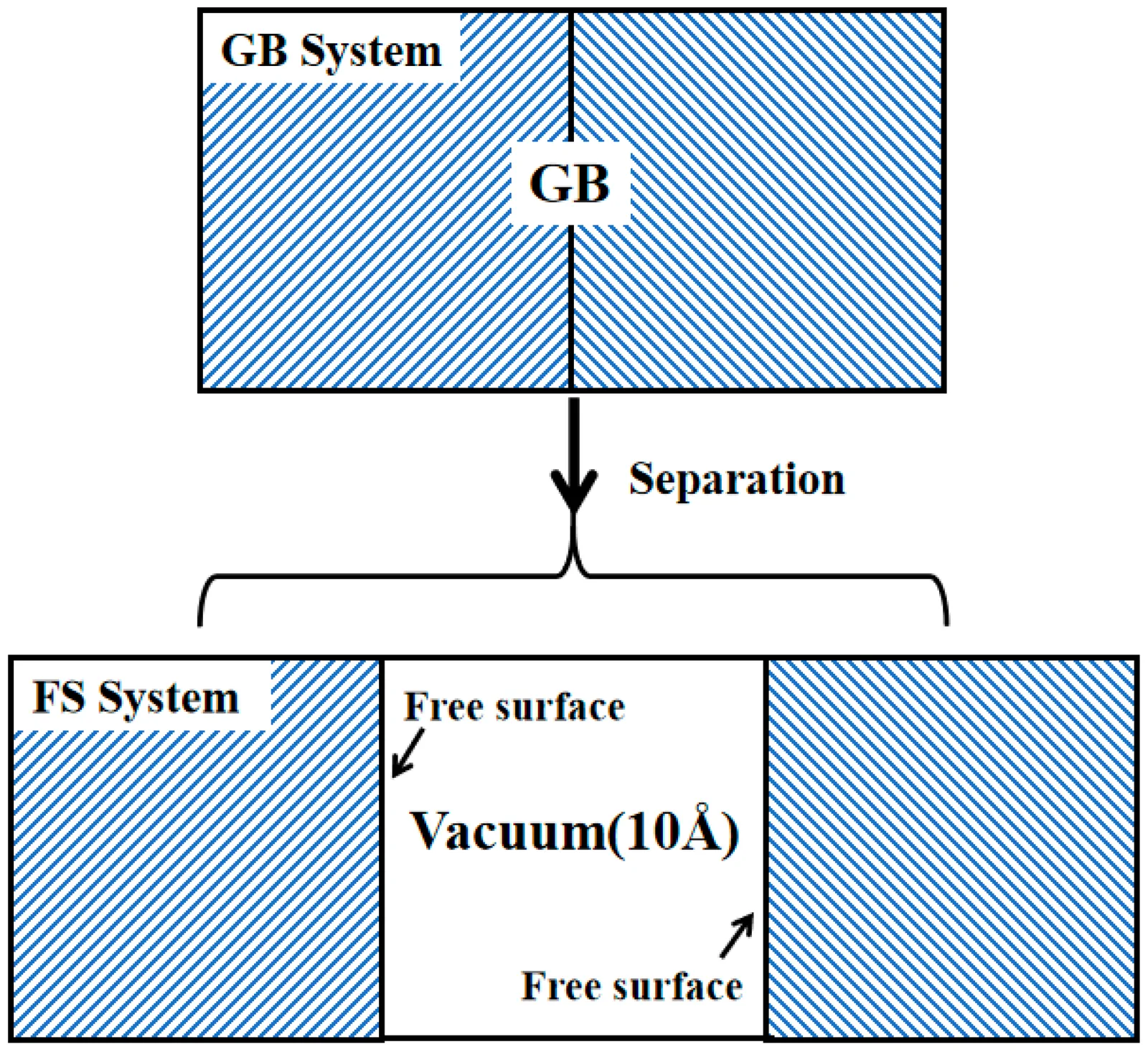
The schematic illustration of Ʃ5(310)/[001] GB separation.

To explore the influence of solute elements X(Y, La, Lu or K) on the toughness and brittleness of W alloys, we calculated the variation characteristics of fracture energy in the W GB system in the presence of solute elements. As shown in Figure 2, the W GB system produces two free surfaces (FSs) after separation and each FS system has two different surfaces. We introduced a sufficiently large vacuum layer between two FSs, with a thickness of 10 Å, to eliminate the interaction between the two FSs. The fracture energy of the Ʃ5(310)/[001] W-GB can be obtained by [4]

(5) $$E_{frac}=\frac{E_{2FS,nX}^{T}{-E}_{GB,nX}^{T}}{S},$$

The total energy of the system containing two FSs and solute atoms (*nX*) after separation is represented by $E_{2FS,nX}^{T}$, the total energy of an unseparated system containing two *FS* and solute atoms is represented by $E_{GB,nX}^{T}$, and the area of the separated crystal interface is represented by *S*. Fracture energy can reflect the tough/brittle characteristics of W GB. A fracture energy greater than zero indicates that the W GBs are strengthened, while a fracture energy less than zero represents that the W GBs are embrittled. The calculation of fracture energy involves relaxation at every stage of the fracture process. The fracture energy of the Ʃ5(310)/[001] W GBs calculated by the above method is 5.185 J/m^2^, and the calculation results are basically consistent with the previous research results (5.150 J/m^2^) [4]. Therefore, the calculation process and model structure we adopted are reasonable.

## 3. Results and Discussion

### 3.1. Solution and Segregation Behavior of Y, La, Lu and K at W Ʃ5(310)/[001] GB

**Figure 3:**
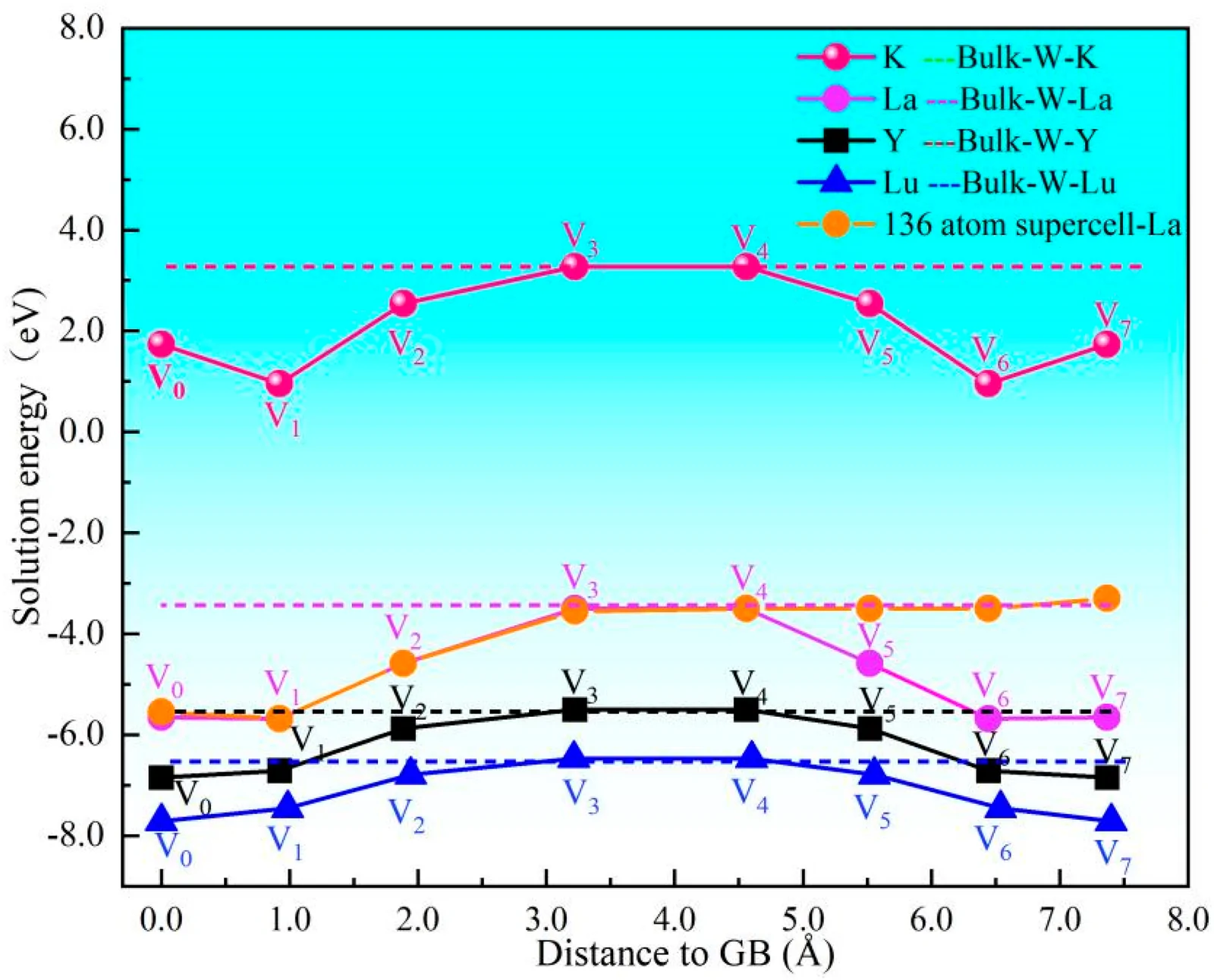
The solution energy of different elements at different substitution sites.

In this study, the solution and segregation behaviors of alloyed elements Y, La, Lu and K in the Ʃ5(310)/[001] W GB region were investigated. As shown in Figure 3, we calculated the solution energies of the Y, La, Lu and K at the substitution sites in the W GB system. The orange line represents the solution energy of La in the 136-atom system, while V_0_–V_7_ represent the substitution sites in the 56-atom system. 0–7 and V_0_–V_7_ correspond to the substitution sites of the 136-atom and the 56-atom system, respectively. The solution energy of La in the 136-atom system is relatively low at sites 0 and 1, gradually increasing as it moves away from the W GB, until site 3 where the solution energy of La tends to stabilize. The indicates that the influence of the W GB on the solution energy of La after site 3 can be almost neglected. In addition, for the convenience of comparison, we also calculated the solution energies of the four elements in the substitution sites of pure W, as indicated by the dashed lines. The solution energies of La at sites V_3_, V_4_, and 3 are almost identical and equal to the solution energy of La in the bulk W, indicating that the 56-atom system is sufficiently large. The solution energies of Y, Lu, and K atoms at V_3_ and V_4_ sites are almost equal to those in bulk W, indicating that the supercell we selected is also reasonable for Y, Lu and K atoms. As shown in Figure 3, Y, La, Lu, and K atoms substitute for the solution energy of substitution sites in the W GB system. The solution energies of La, Lu, and K atoms at V_3_ and V_4_, V_2_ and V_5_, V_1_ and V_6_, and V_0_ and V_7_ sites are equal, due to the symmetric structure of the 56-atom W GB system. The solution energy of all four elements is low near the W GB region, and increases as they move away from the W GB until they approach the solution energy in bulk W. The solution energies of Y La and Lu metal atoms at substitute (Sub) sites in the W GB system are both less than zero, indicating that it is relatively easy for them to stably occupy Sub sites. The solution energy of K atoms at the Sub site in the W GB system is greater than zero, indicating that K requires more energy to dissolve at that site, due to the large difference in radius between K and W atoms. The results show that the solution energy of these alloying elements is low near the W GB, which indicates that the W GB has a remarkable trapping ability.

As shown in Figure 3, comparative analysis reveals that the solution energy of K atoms is the highest and the solution energy of Lu atoms is the lowest at the same substitution site in the 56-atom system, due to the different atomic radii of the four alloying atoms. Specifically, the order of atomic radii of the four alloys from large to small is K > La > Y > Lu [15,45]. Therefore, at the same site in the 56-atom W GB system, the order of solution energies for the four alloy elements from large to small is K > La > Y > Lu. The solution energy is not only related to the interactions between atoms, but also to the atomic radius. A larger atomic radius requires more space for solution, which in turn requires more energy. As shown in Table 1, we not only calculated the solution energies of K, La, Y, and Lu in the W GB region, but also calculated the solution energies of the four alloying atoms in bulk W as Sub, tetrahedral interstitial sites (TISs), and octahedral interstitial sites (OISs). The results indicate that all four atoms have the lowest solution energy at substitution sites in the bulk W, and the highest solution energy at octahedral interstitial sites.

**Figure 4:**
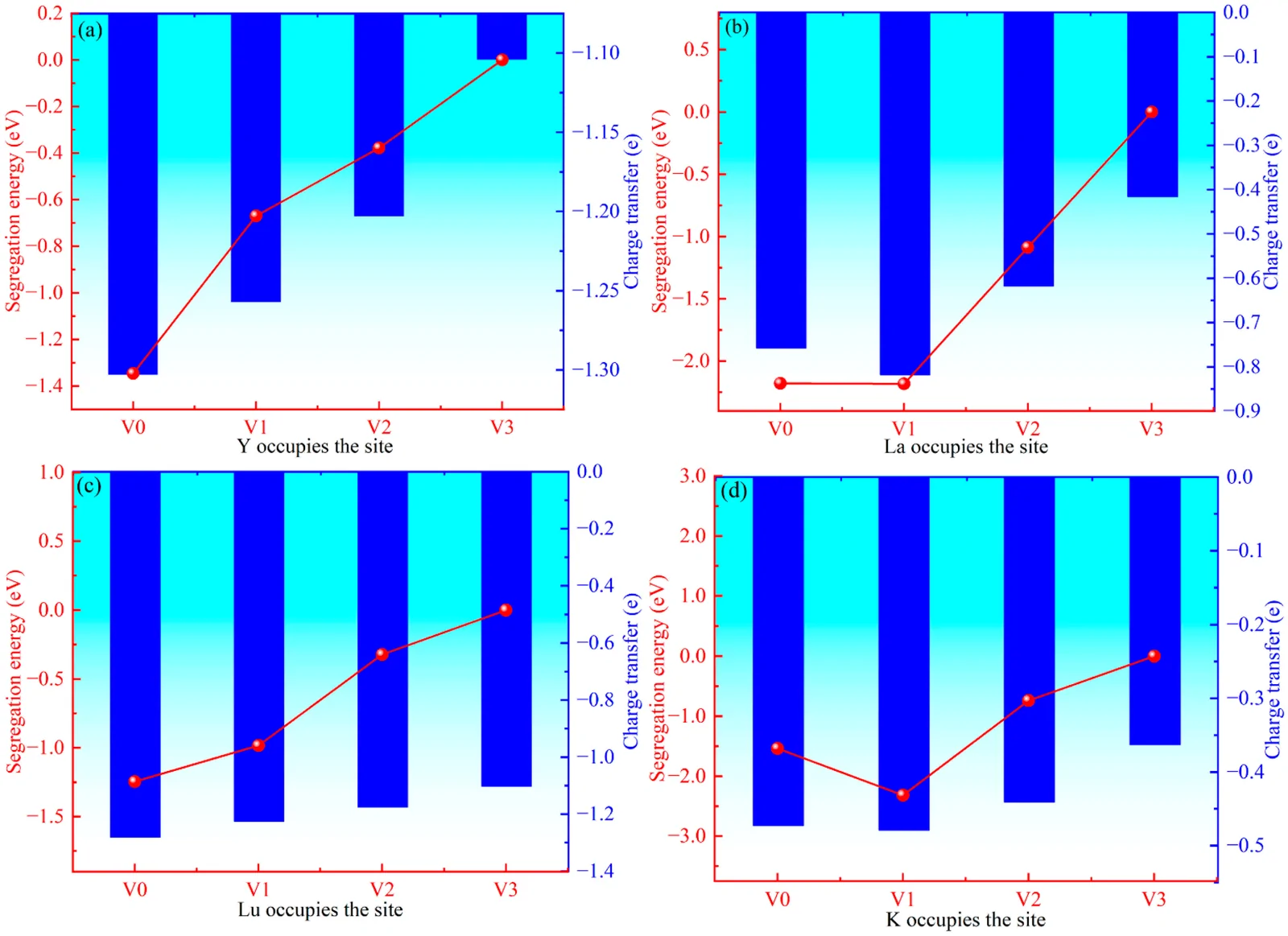
Segregation energy and Bader charge of Y, La, Lu, and K at V_0_~V_3_ sites. (**a**–**d**) denotes Y, La, Lu and K at V_0_~V_3_ sites, respectively.

The addition of alloying elements Y, La, Lu or K can improve the toughness of W-based materials, because they can affect the strength and toughness of W GB. Therefore, the segregation of alloying elements in the W GB region is very important [24,26]. To illustrate the influence of W GB on Y, La, Lu, and K atoms, we also calculated the segregation energy and Bader charge of Y, La, Lu, and K at different substitution sites as shown in Figure 3 and Table 2. The segregation energies of La, Lu, and K at V_0_~V_3_ sites are all less than zero, indicating that these four atoms tend to occupy the sites in the W GB region. As shown in Figure 4, the segregation energies of the four elements at V_1_~V_3_ sites are all less than zero. Specifically, the segregation energies of Y and Lu atoms at the V_0_~V_3_ sites increase with increasing distance from the W GB system. In addition, La and K atoms have the lowest segregation energy at the V_0_~V_3_ site, and there is a difference in segregation energy between them and the other two metal atoms in the W GB system. Atomic radius can affect the solubility energy of atoms and the metal bond between solute atoms and tungsten, and charge transfer can also affect the stability of solute atoms in the matrix. Analyzing the Bader charge in Figure 4, it was found that the segregation energies of the Y, La, Lu, and K atoms are proportional to the charge transfer, indicating that the W GB region can promote the interaction between W and alloy elements, and the charge transfer promotes segregation. The charge transfer between atoms can reflect the strength of the interaction between atoms and surrounding atoms. According to Table 2, the amount of charge transfer between Y, La, Lu, and K in the W GB system can better reflect the interaction relationship between alloy atoms and W. Y, La, Lu, and K all lose electrons in the W GB system, and the amount of electron loss is positively correlated with segregation energy. It should be noted that the segregation energies of Y and Lu at the V_0_ site are −1.346 eV and −1.246 eV, with the maximum segregation energy relative to the V_1_~V_3_ sites. At this point, the corresponding charge transfers are −1.303 e and −1.281 e, respectively. In addition, the most stable sites for La and K are V_1_, with segregation energies of −2.179 eV and −1.539 eV. When La and K atoms occupy the V_1_ site in the 56-atom W GB system, they lose electrons of −0.818 e and −0.479 e, respectively. By comparison, it is found that the most stable occupation site of Y and Lu in the 56-atom W GB system is the V_0_ site on the W GB interface, while the stable occupation site of La and K atoms is the V_1_ site nearest to the W GB interface.

**Figure 5:**
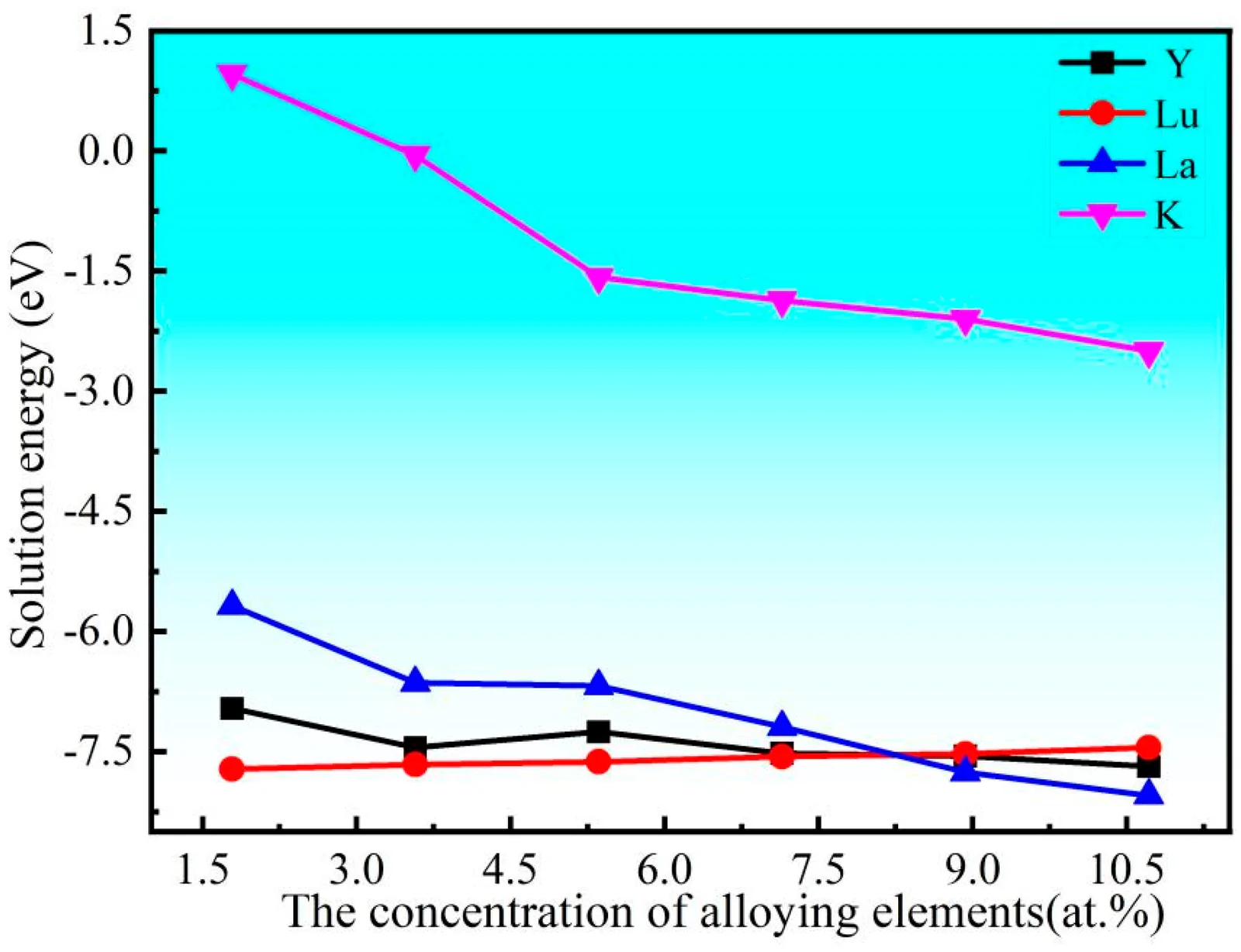
Relationship between alloyed element content and solution energy of individual atom.

The results show that the aggregation state of K and Y elements at W GB will affect the strength of W GB [4,15]. It is of great significance to study the aggregation state of alloying elements at W GB for the development of strong and tough W alloys. We systematically calculated the solution of alloying atoms (Y, La, Lu and K) in the W GB region and the effect on the strength and toughness of the W GB with the increase in alloying element content. As shown in Figure 5, the solution energy of a single atom in the W GB region can be obtained by adding alloyed atoms to the W GB region one by one. The results in Figure 5 show that the solution characteristics of K and La in the W GB region are similar: with the increase in alloy element content, the solution energy of a single (K or La) atom gradually decreases. The phenomenon can be attributed to the strong attraction between K and K and between La and La in the W GB region. The self-attraction causes the solution energy of K and La introduced later to decrease sharply, thus promoting their solution in the W GB region. In contrast, the solubility characteristics of Y and Lu in the W GB region are consistent. The alloying atoms existing in the W GB system have little effect on the solution energy of the subsequent introduced atoms (Y or Lu), and sometimes even lead to the increase in the solution energy of the introduced atoms.

### 3.2. Strengthening Mechanism of Y, La, Lu and K on Tungsten Ʃ5(310)/[001] Grain Boundary

**Figure 6:**
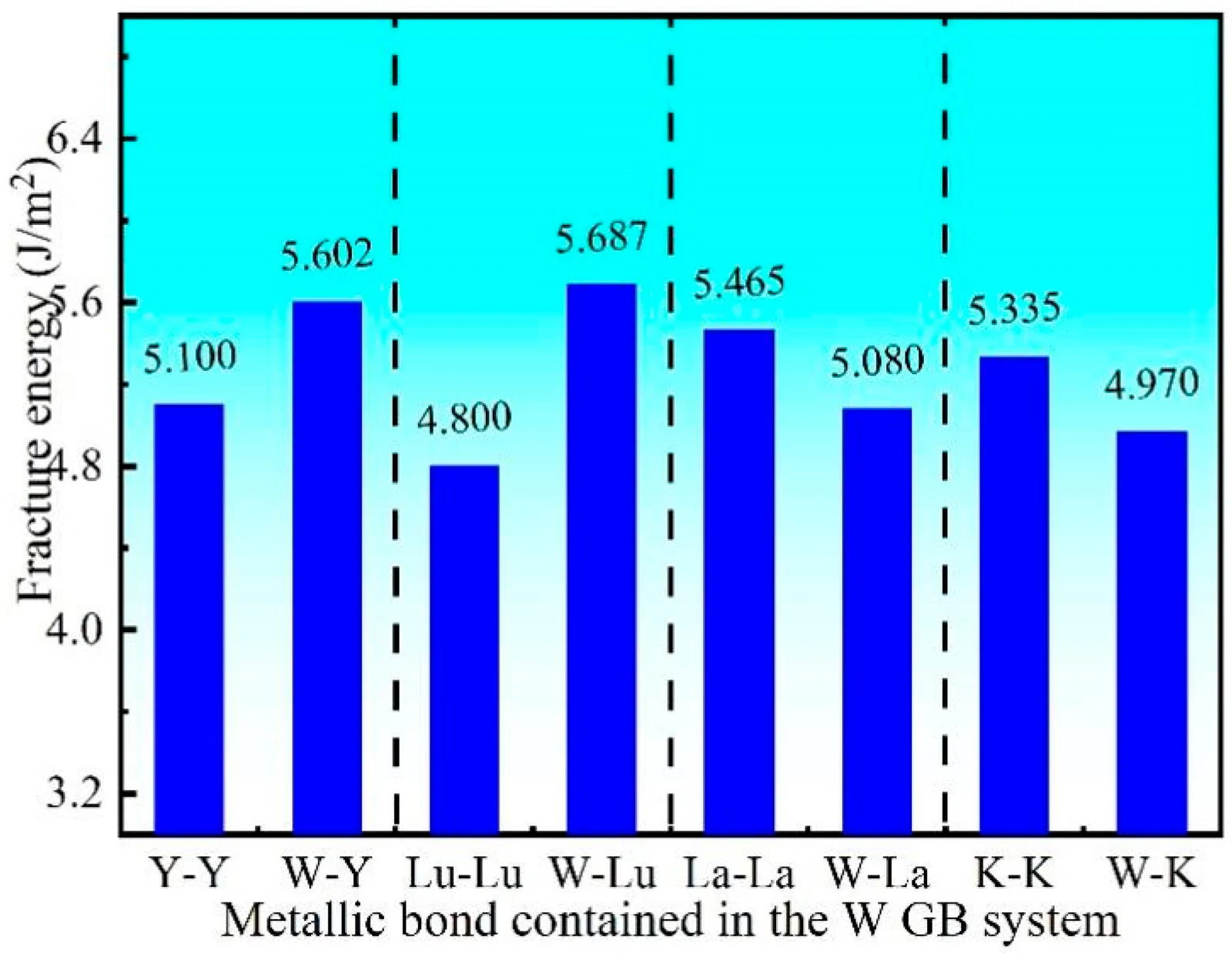
Fracture energy of W GB in the presence of different metal bonds.

In our previous study, we obtained the influence characteristics of K and Y on GB strengthening and toughening through fracture energy. The effect of K and Y on the strengthening and toughening of W GB has been explored. The results show that the reason for the strengthening of W GB by alloying elements K and Y is the formation of strong metal bonds K-K and W-Y [15,24]. We have systematically analyzed the effect of alloying elements (Y, La, Lu and K) on the fracture of Ʃ5(310)/[001] W GB. It is found that Y, La, Lu and K can enhance the W GB strength. As shown in Figure 6, the fracture energy of W GB shows an obvious difference in the presence of different metal bonds. When Y is added to W, two types of metallic bonds are formed: Y-Y metallic bonds and W-Y metallic bonds. When the Y-Y metal bond is broken, the fracture energy of W GB is 5.100 J/m^2^, while when the W-Y metal bond is broken, the fracture energy is 5.602 J/m^2^ [24], which is higher than that of pure W GB 5.185 J/m^2^. Therefore, it can be considered that the strengthening effect of Y on W GB is mainly dependent on the strong W-Y metal bond formed by it.

Similarly, when Lu is added to W, the fracture energy of W GB is affected by the Lu-Lu metal bond and W-Lu metal bond. The breaking energy of the metal bond between Lu and Lu is 4.800 J/m^2^, while the breaking energy of the metal bond between W-Lu is 5.687 J/m^2^. This shows that the strengthening effect of Lu on W GB is mainly due to the strong W-Lu metal bond formed between Lu and W. In addition, we also studied the strengthening mechanism of W GB by La and K. The results show that when La is added to W, the fracture energy of the La-La metal bond is 5.465 J/m^2^, which exceeds the fracture energy of pure W GB. In this case, La-La metal bonds play a key role in W GB strengthening. The study on K shows that the fracture energy of K-K and W-K metal bonds destroyed by W GB fracture is 5.335 J/m^2^ and 4.970 J/m^2^ [15], respectively. In summary, alloying elements K and La improve the properties of W GB because of the formation of strong metal bonds K-K and La-La, while Y and Lu have similar strengthening effects on W GB by forming strong metal Y-W and Lu-W bonds. In addition, the change in charge density and bond length between atoms can reveal the strengthening mechanism of alloying elements (Y, La, Lu and K) on W GB at the atomic level.

**Figure 7:**
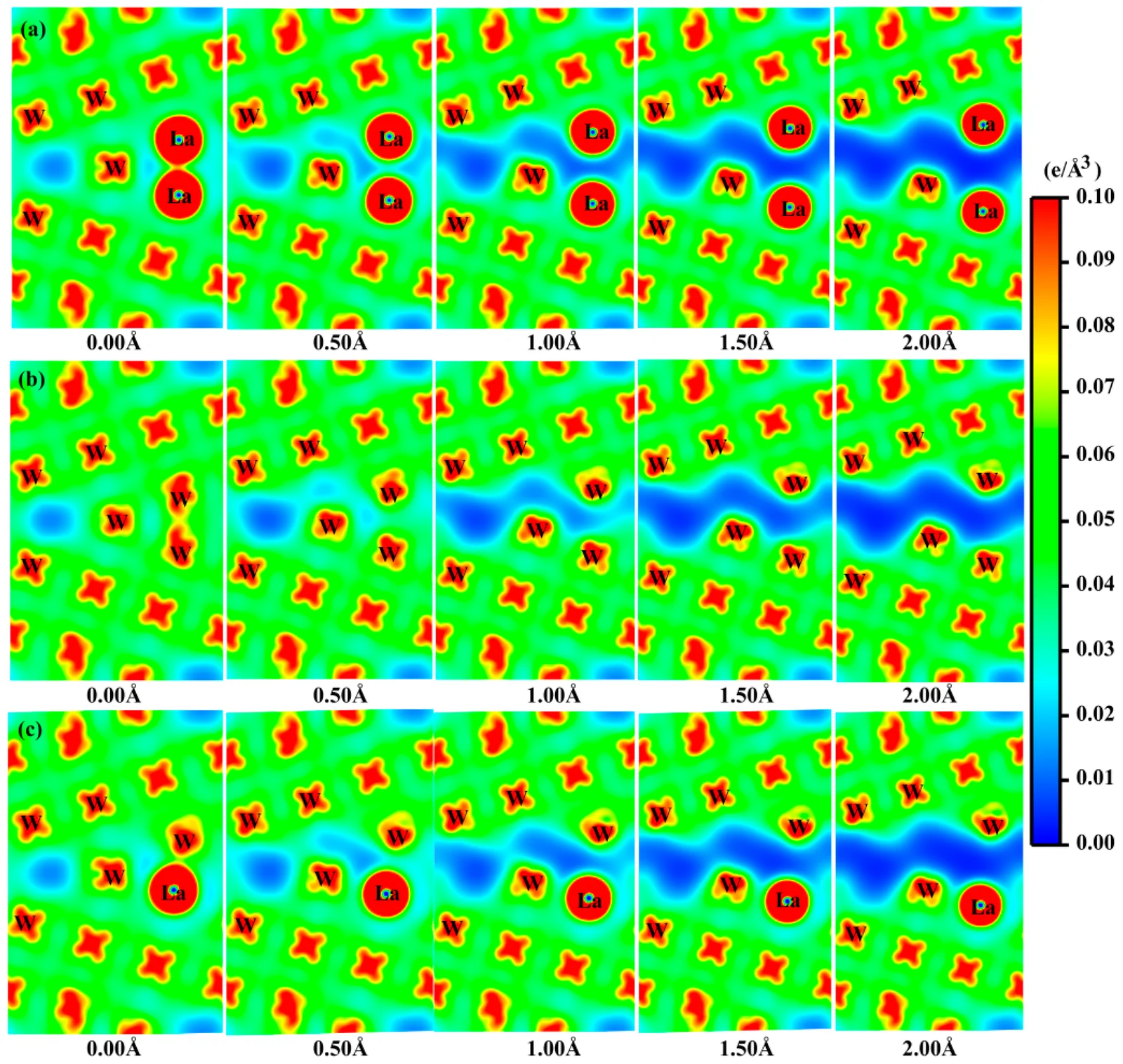
Schematic illustrations of the charge density evolution in the Σ5(310)[001] W GB planes (**a**) with two La atoms at V_1_ and the symmetry site of V_1_, (**b**) without a La atom, and (**c**) with a La atom at V_1_.

In order to analyze the internal mechanism of the strengthening effect of alloying elements on the W GB, we have analyzed the relationship between the change in charge density of the La element in the W GB region and the W GB strengthening in detail. It can also be found from the charge density that the La element can form a strong metal La-La bond in the W GB region. Figure 7a shows the charge density diagram when La occupies the V_1_ site and the V_1_ symmetrical site, Figure 7b shows the charge density diagram of W GB, and Figure 7c shows the charge density diagram when La occupies the V_1_ site. The charge density diagram shows that the interaction between La-La bonds is the strongest, the interaction between La-W bonds is the weakest, and the strength of W-W metal bond is between La-La and La-W. This is consistent with the results that the separation energies of La-La, W-W and La-W bonds in W GBs are 5.465 J/m^2^, 5.185 J/m^2^ and 5.080 J/m^2^ respectively. Similarly, we have analyzed in detail the relationship between the bond length change of the Y element in the W GB region and the mechanical properties of W GB. The results show that the increase in fracture energy in the W GB region is due to the enhancement of W-Y bonds [17]. However, there is no systematic comparative analysis of the relationship between the strengthening and toughening mechanisms of Y, La, Lu and K in the W GB region. Mohammed et al. [46] obtained the mechanical properties and changes in mechanical properties of perovskite by analyzing its electronic structure, so it is necessary to analyze in detail the electronic structure changes of alloying elements in the W GB system.

**Figure 8:**
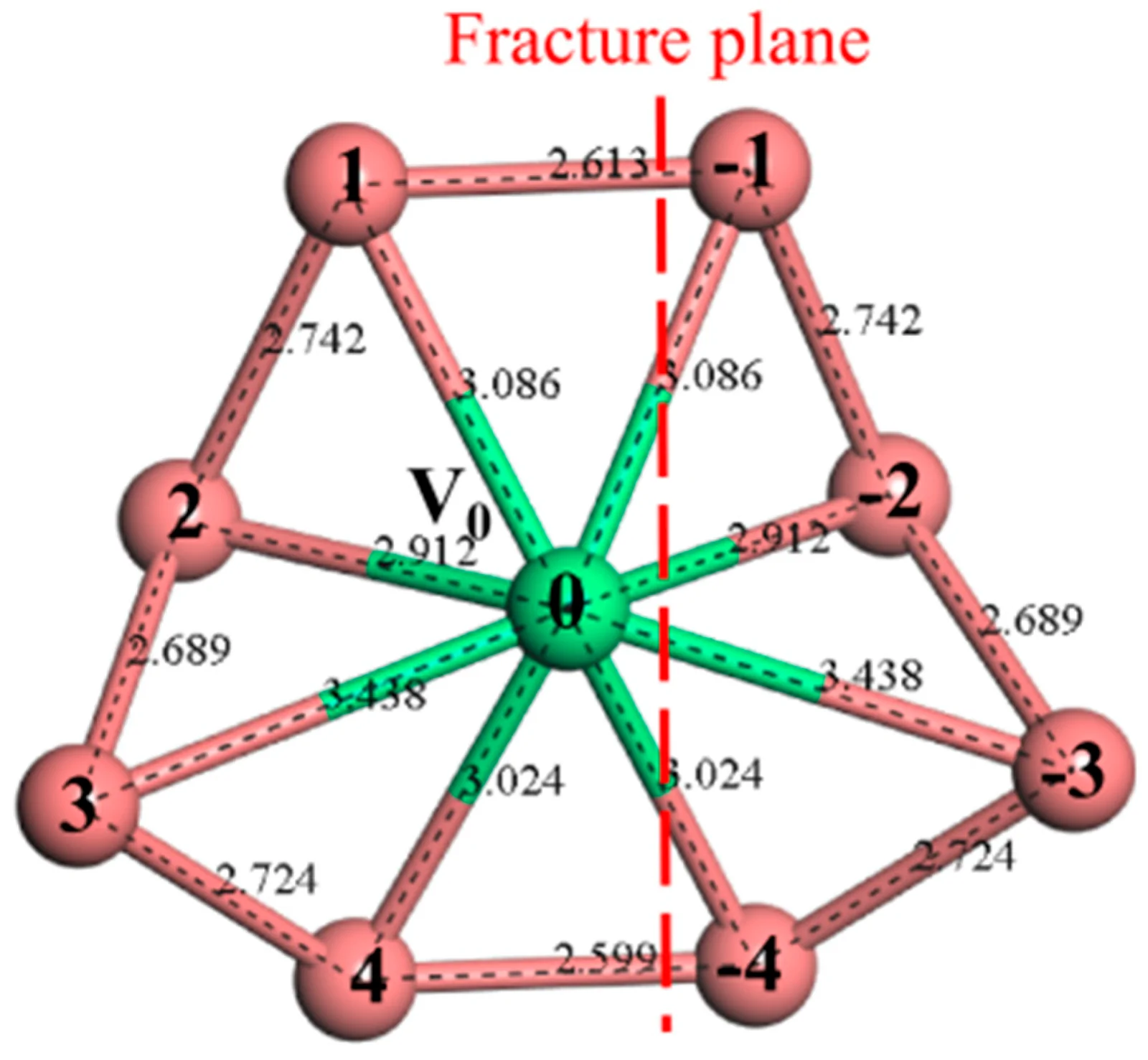
Bond lengths around the W GB plane for W GB with a segregated Lu atom at site V_0_. The brown and green balls represent W and Lu atoms respectively. The numbers represent different sites occupied by W or Lu atoms.

The research results indicate that Y, La, Lu and K can strengthen the W GB, because La and K form strong La-La and K-K bonds in the W GB region, and Y forms Y-W metal bonds in the W GB region. Next, we explored in detail the effect of alloying element Lu on the strengthening and toughening properties of W GB. Physically, fracture energy is determined by the strength of the interaction between atoms [4,43]. Figure 8 shows the metal bond length around a Lu atom at the V_0_ site in the Ʃ5(310)/[001] W GB system, and the red dashed line represents the fracture path set for calculating the fracture energy. As shown in Figure 8, when Lu occupies the V_0_ site of the W GB, it is found that the W GB system has good mirror symmetry based on the bond length between Lu and surrounding atoms. Similarly, Wu et al. [24] showed that the clean W GB also has good mirror symmetry. Furthermore, Wu et al. described in detail the metal bond length between atoms in the stable clean W GB region. The results show that [24] breaking the clean W GB entails the breaking of the W(1)-W(−1) bond, W(0)-W(−1) bond, W(0)-W(−2) bond, W(0)-W(−3), W(0)-W(−4) bond and W(4)-W(−4) bond. The lengths of W(1)-W(−1) and W(4)-W(−4) bonds in clean GB are shorter than the other four metal bonds, and hence contribute the most to clean GB separation. As can be seen in Figure 8, if a W atom is replaced by Lu in site V_0_, the lengths of W(1)-W(−1), W(4)-W(−4), Lu(0)-W(−2), Lu(0)-W(−3) and Lu(0)-W(−4) bonds both increase, but only the bond lengths of Lu(0)-W(−1) decrease for clean W GB. Therefore, adding Lu atoms to the W GB system can increase the W GB strength due to the formation of strong Lu-W metal bonds.

**Figure 9:**
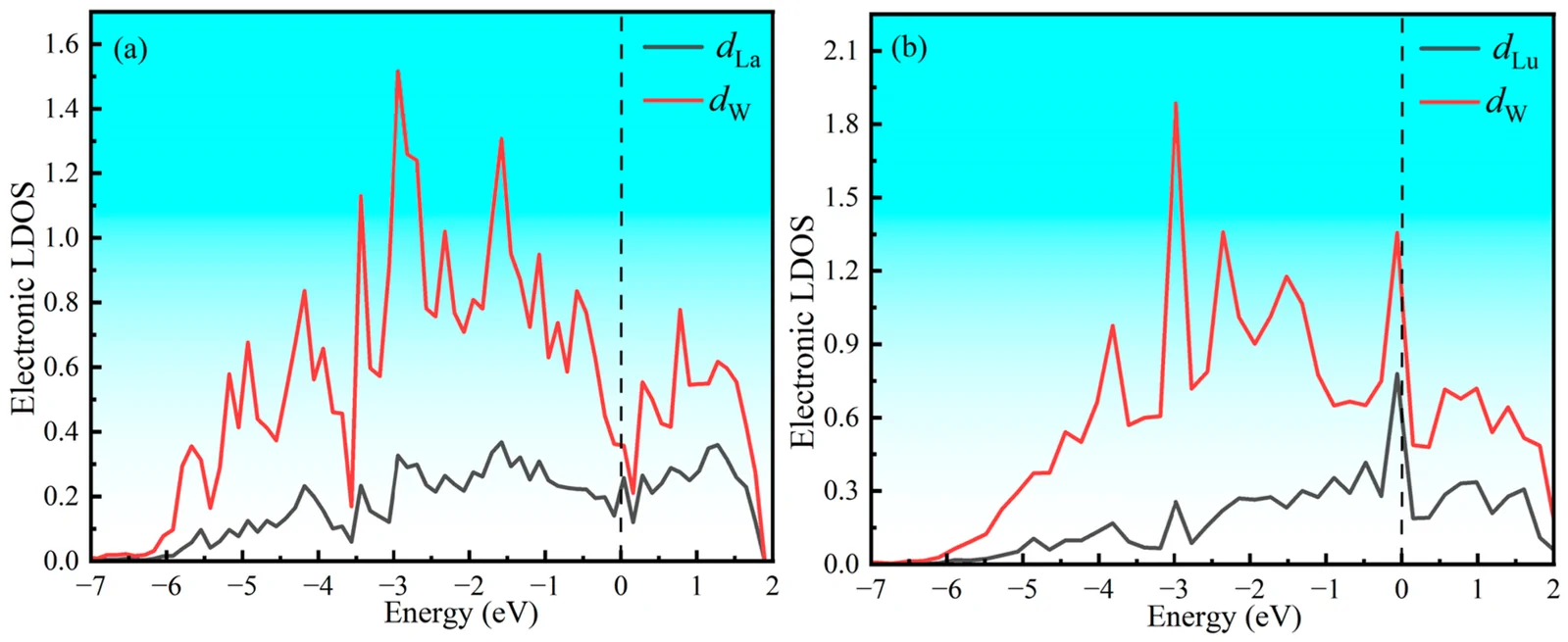
Local electronic density of states (LDOS) of W-d, La-d and Lu-d orbitals in W GB system: (**a**) La occupies the *d* orbitals of the V_1_ site and V_1_ symmetric site W; (**b**) Lu occupies the *d* orbitals of W atoms at the V_0_ and −1 sites.

As is well known, the local electronic density of states (LDOS) can reflect the strength of the interaction between two atoms. As shown in Figure 9a, we calculated the density of *d* orbital electronic states of W occupied by La at V_1_ and V_1_ symmetric sites in the W GB system. As shown in Figure 9b, the electron density of *d* orbitals occupied by Lu at the V_0_ site and W at the −1 site is represented. La occupies the V_1_ site and the W atom closest to the V_1_ symmetric site, and the interaction between La and W is crucial during the fracture process. Similarly, the W at the V_0_ and −1 sites occupied by Lu serves as a reinforcing metal bond during the fracture process. Comparison reveals the presence of two strong hybridization peaks between Lu and tungsten around −3 eV and 0 eV, indicating a strong interaction between W and Lu. On the contrary, the hybridization degree between La and W is relatively low. The analysis of LDOS results shows that the strengthening of W GBs by Lu is caused by strong W-Lu bonds, which is consistent with the analysis of fractured metal bonds.

## 4. Conclusions

This paper comprehensively investigates the dissolution and segregation behaviors of alloying elements (Y, La, Lu, and K) at the Ʃ5(310)/[001] W GB. Furthermore, the influence mechanism of four elements on the strength of W GB was analyzed. The results show that these elements have low solution energy and segregation energy in the W GB region, indicating that the W GB has a strong ability to capture the four alloy elements. The solution energies of the four metal elements at the same substitution sites at the W GB are K > La > Y > Lu in descending order, because the atomic radius of the alloy elements is K > La > Y > Lu in descending order. Therefore, the atomic radius is the main factor affecting the dissolution energy of alloying elements at the W GB. The electronic structure analysis showed that the segregation energies of the four metal elements at the W GB were positively correlated with the number of electron transfers. It is worth noting that the most stable occupation sites of La and K in the W GB region are the sites (V_1_) nearest to the GB plane, and the most stable occupation sites of Y and Lu in the W GB region are the sites (V_0_) on the W GB plane. The presence of K or La atoms in the W GB region will promote the dissolution of subsequent alloy atoms. On the contrary, the presence of Y or Lu in the W GB region does not significantly promote the dissolution of subsequent alloy atoms. The reason is that there is a strong attraction between K and K (La and La), while the interaction between Y and Y (Lu and Lu) is relatively weak. The mutual attraction between alloying atoms also has an important effect on the mechanical properties of W GB. In terms of mechanical properties, the addition of alloying elements significantly improves the fracture energy of W GB, and the Y and Lu effectively increase the fracture energy to 5.602 J/m^2^ and 5.687 J/m^2^, which is higher than the 5.185 J/m^2^ of clean W GB. This strengthening mechanism is mainly attributed to the strong metal bond between W-Y and W-Lu. Although the fracture energy of La and K is also higher than that of clean W GB—5.465 J/m^2^ and 5.335 J/m^2^, respectively—the strength improvement mainly depends on the action of La-La and K-K metal bonds. In summary, this study reveals the potential of rare earth elements in improving the properties of W-based alloys, and provides important theoretical guidance for the design of high-performance W alloy materials. The influence of different rare earth and K element contents on the toughness and brittleness characteristics of W alloys can be studied through experiments, and the combined effect of rare earth and K elements on the service performance of W-based alloys for nuclear fusion can be explored.

## Figures and Tables

**Table 1:** Solution energy (eV/atom) of Y, La, Lu, and K at different sites in W GB and bulk W.

Elements	V_0_	V_1_	V_2_	V_3_	Sub	TIS	OIS
Y [24]	−6.848	−6.712	−5.880	−5.502	−5.579	9.756	10.999
La	−5.656	−5.679	−4.582	−3.495	−3.551	10.220	12.320
Lu	−7.717	−7.454	−6.793	−6.471	−6.490	9.271	10.283
K [15]	1.739	0.959	2.539	3.278	3.328	15.220	16.639

**Table 2:** Segregation energy *E*^seg^ (eV) and Bader charge (e) of Y, La, Lu, and K at substitution sites in the W GB system.

Elements	V_0_		V_1_		V_2_		V_3_	
	*E* ^seg^	Bader Charge	*E* ^seg^	Bader Charge	*E* ^seg^	Bader Charge	*E* ^seg^	Bader Charge
Y [24]	−1.346	−1.303	−0.670	−1.257	−0.378	−1.203	0.077	−1.104
La	−2.179	−0.758	−2.184	−0.818	−1.087	−0.618	0.056	−0.416
Lu	−1.246	−1.281	−0.983	−1.225	−0.322	−1.175	0.019	−1.102
K [15]	−1.539	−0.473	−2.319	−0.479	−0.739	−0.441	0.050	−0.363

## Data Availability

The original contributions presented in this study are included in the article. Further inquiries can be directed to the corresponding authors.
